# Carvacrol as a Therapeutic Candidate in Breast Cancer: Insights into Subtype-Specific Cellular Modulation

**DOI:** 10.3390/biology14101443

**Published:** 2025-10-19

**Authors:** Asmaa Abuaisha, Emir Nekay, Ozgur Yilmaz, Baris Yildiz, Tarik Mecit, Cuneyd Yavas, Berrin Papila, Halil Ibrahim Arslan, Aybuke Hilal Gumus, Esra Nazligul, Sadiye Akbas, Selman Emiroglu

**Affiliations:** 1 Biruni Research Center B@MER, Biruni University, 34015 Istanbul, Turkey; 2 Department of General Surgery, Faculty of Medicine, Biruni University, 34015 Istanbul, Turkey; 3 Department of Internal Medicine, Kanuni Sultan Suleyman Training and Research Hospital, 34303 Istanbul, Turkey; 4 Life Sciences and Technology Implementation and Research Center, Kafkas University, 36000 Kars, Turkey; 5 Department of Physiology, Faculty of Medicine, Biruni University, 34015 Istanbul, Turkey; 6 Department of Molecular Biology and Genetics, Biruni University, 34015 Istanbul, Turkey; 7 Department of General Surgery, Cerrahpaşa Faculty of Medicine, Istanbul University-Cerrahpaşa, 34320 Istanbul, Turkey; 8 Department of Molecular and Medical Genetics, Graduate School of Education, Biruni University, 34015 Istanbul, Turkey; 9 Department of Internal Medicine, Division of Hematology, Istanbul Faculty of Medicine, Istanbul University, 34015 Istanbul, Turkey; 10 Department of General Surgery, Division of Breast Surgery, Istanbul Faculty of Medicine, Istanbul University, 34015 Istanbul, Turkey

**Keywords:** breast cancer, carvacrol, apoptosis, ROS, CD44^+^, gene expression, BC treatment

## Abstract

**Simple Summary:**

This study investigated the anticancer effects of carvacrol, a natural compound, in two breast cancer cell types (HR^+^ MCF-7 and TNBC MDA-MB-231). Carvacrol reduced cell growth and migration, increased apoptosis by raising the BAX/BCL2 ratio, and lowered ROS levels, showing stronger antioxidant effects in MCF-7 cells. It also decreased CD44^+^ stem cell marker expression but did not affect CD133^+^. Gene analysis revealed subtype-specific differences: *ABCG2* increased in MCF-7 but decreased in MDA-MB-231, while *NF_K_B1* rose in both. Overall, carvacrol shows multiple anticancer actions and may be a promising natural option for breast cancer treatment, though more in vivo and clinical studies are needed.

**Abstract:**

Background: Carvacrol, a natural phenolic monoterpenoid, has been suggested to exert anticancer effects; however, its underlying mechanisms in breast cancer (BC) remain incompletely defined. Methods: MCF-7 (HR^+^) and MDA-MB-231 (TNBC) BC cell lines were treated with carvacrol at various concentrations. Cell viability was assessed using CVDK8 kit, while migration was evaluated by wound healing assays. Apoptosis was determined using Annexin V-FITC Kit, and ROS levels were measured by DCFH-DA assay. Flow cytometry was used for CD44/CD133 cancer stem cells markers analysis, and genes expression were quantified using qPCR. Results: Carvacrol significantly inhibited cell proliferation, and migration in both HR^+^ and TNBC cells. Additionally, carvacrol increased the *BAX/BCL2* ratio, induced apoptosis, and decreased ROS levels, with greater antioxidant activity observed in MCF-7 cells. Moreover, carvacrol suppressed CD44^+^ levels, whereas CD133^+^ levels were not affected. Gene expression analysis revealed subtype-specific effects where *ABCG2* was upregulated in MCF-7 cells but downregulated in MDA-MB-231 cells, while *NF_K_B1* expression increased in both lines. Conclusions: Carvacrol exerts multitargeted anticancer effects in BC by promoting apoptosis, reducing ROS, and suppressing CD44^+^, with distinct subtype-specific responses. These findings highlight carvacrol as a promising natural therapeutic compound for BC treatment; however, further in vivo studies and clinical investigations are required to validate its translational potential.

## 1. Introduction

Breast cancer (BC) is one of the most diagnosed malignancies among women worldwide, and accounted 11.6% of all cancer incidence cases, and 6.9% of all cancer-related deaths [1]. BC is generally classified into four major molecular subtypes: luminal A, luminal B, HER2-enriched, and triple-negative breast cancer (TNBC) [2]. BC demonstrates marked biological heterogeneity at both the intertumoral and intratumoral levels, which plays a central role in determining the clinical outcomes [3]. Advancements in screening strategies and treatment have markedly improved the clinical management and survival outcomes of BC in recent decades [4]. Nevertheless, prolonged administration of standard therapies frequently results in adverse drug reactions, toxicity, and the development of resistance [5]. In this context, the development of novel agents capable of enhancing both the safety and efficacy of oncological treatments represents not only an unmet clinical need but also a strategic priority in modern cancer care [6,7].

The proposition that phytotherapeutic or herbal extract applications could be utilized in cancer treatment due to their high antioxidant content has become a highly debated topic over the past two decades [8,9]. In the 1990s, antioxidants were often perceived as universal remedies, a view that also influenced certain non-scientific attempts to promote phytotherapy as an alternative to conventional cancer treatments [10]. However, accumulating evidence suggests that a high antioxidant content may, in some cases, impair the body’s ability to combat cancer cells and diminish the apoptotic efficacy of chemotherapy and radiotherapy [11,12]. These considerations underscore the importance of carefully characterizing plant-derived compounds and elucidating their mechanistic effects, rather than relying on generalized assumptions, thereby positioning phytochemicals as potential anticancer candidates within a rigorous scientific framework [13].

Carvacrol is a monoterpenoid phenolic compound obtained primarily from plants such as oregano (Origanum) and thyme (Thymus), renowned for its anti-microbial, anti-inflammatory, antioxidant and anti-angiogenic properties [14,15]. Additionally, it displays anticancer and anti-inflammatory properties by inducing apoptosis, decreasing oxidative stress, and modulating cell-cycle progression, thereby suppressing tumor growth. Furthermore, carvacrol inhibits migration, invasion, and angiogenesis, and exerts regulatory effects on critical oncogenic signaling cascades, including PI3K/AKT/mTOR, MAPK, STAT3, and Notch [16]. However, studies have revealed that carvacrol also exerts anti-proliferative and pro-apoptotic effects on BC cells [17,18]. Moreover, carvacrol has been reported to suppress the *PI3K/Akt* signaling pathway, facilitating cell cycle arrest (G0/G1 phase accumulation) and activation of pro-apoptotic proteins in BC cells [19].

This study aimed to elucidate the anticancer potential and subtype-specific biological effects of carvacrol on BC by comparatively analyzing HR^+^ (MCF-7) and TNBC (MDA-MB-231) cells. Unlike previous studies that focused on single cell models or limited endpoints, our research provides a comprehensive evaluation of apoptosis, oxidative stress and stemness related pathways across two distinct BC phenotypes, thereby revealing novel insights into the mechanistic diversity of carvacrol’s action.

## 2. Materials and Methods

### 2.1. Selection of Genes and Public Database-Based Evaluation of Genes Expression and Survival Effects in Breast Cancer Patients

*BAX* (Bcl-2 Associated X-protein), a pro-apoptotic gene, and *BCL2* (b-cell lymphoma 2), an anti-apoptotic gene, are considered key markers of apoptotic stimuli. The expression ratio (*BAX*/*BCL2*) reflects the apoptotic tendency of cells. *NF_K_B1* (Nuclear Factor Kappa B Subunit 1) is a multifunctional transcription factor expressed in nearly all cell types, acting as the final target of diverse signaling cascades. These pathways are activated by numerous stimuli and regulate a broad spectrum of biological processes, including inflammation, cellular differentiation, proliferation, tumor development, and programmed cell death. *ABCG2* (ATP-Binding Cassette Sub-Family G Member 2) gene is a member of the ATP-binding cassette (ABC) family of efflux transporters. *ABCG2* was first identified in a multidrug-resistant BC cell line, where its expression was shown to mediate resistance against several chemotherapeutic drugs. These genes were chosen to investigate the effect of carvacrol on BC cells because they represent critical regulators of apoptosis (*BAX*, *BCL2*), transcriptional control of tumor-related processes (*NF_K_B1*), and drug resistance mechanisms (*ABCG2*), and also the protein–protein interaction (PPI) network among them shows strong connectivity.

The Cancer Genome Atlas (TCGA) database (https://cancergenome.nih.gov/ (accessed on 20 January 2025)) [20], a comprehensive resource of genomic and transcriptomic profiles across various cancer types, was used to retrieve Breast Cancer (BRCA) expression data. We compared the expression levels of *BAX*, *BCL2*, *NF_K_B1*, and *ABCG2* genes between 114 normal tissue samples and 1097 primary tumor samples from this dataset. The ClustVis web tool (https://biit.cs.ut.ee/clustvis/ (accessed on 21 January 2025)) was then utilized to perform principal component analysis and generate heatmaps, allowing the visualization of expression patterns and hierarchical clustering among the samples [21].)

The effect of the studied genes on the overall survival and disease-free survival in BC patients were defined using the Kaplan–Meier plotter (via GEPIA2) (http://gepia2.cancer-pku.cn/ (accessed on 21 January 2025)) [22,23].

The STRING database (https://string-db.org (accessed on 22 January 2025)) [24] was used to evaluate the interactions among the identified genes. The official gene names were input into the search field, and interaction networks were generated accordingly.

### 2.2. Cell Lines and Cell Culture

The human MCF-7 hormone receptor-positive (HR^+^ BC), and MDA-MB-231 (TNBC) cell lines were cultured in high glucose Dulbecco’s Modified Eagle Medium (DMEM) (EcoTech Biotechnology, Erzurum, Turkey) supplemented with 10% fetal bovine serum (FBS) (Gibco, London, UK), and 1% antibiotic (penstrep) (Gibco, UK). Cells were incubated in an incubator (Wiggens WCI-180, Wuppertal, Germany) with humidity >90% and 5% CO_2_ at 37 °C.

### 2.3. Carvacrol Preparations

Carvacrol (99.56% purity) (Ambeed, Buffalo Grove, VA, USA) was dissolved in DMSO (EcoTech Biotechnology, Erzurum, Turkey) (200 mM stock) and stored at room temperature. Cells were treated with 300–1000 μM carvacrol for 48 h to determine IC_50_ values, which were used in subsequent assays.

### 2.4. Cell Viability

MCF-7 and MDA-MB-231 cells were seeded in quadruplicate (4 × 10^3^ cells/well) in 96-well plates (Nest Biotechnology, Wuxi, China) and incubated for 24 h (*n* = 2). Cells were treated with 300–1000 μM carvacrol. Non-treated cells were used as a negative control. After 48 h of carvacrol treatment, 10 μL of cell viability detection kit 8 (CVDK8) solution (EcoTech Biotechnology, Erzurum, Turkey) was added to each well and cells were incubated for 3 h in a CO_2_ incubator. Absorbances was measured at 450 nm using microplate Reader (BioTek, Shoreline, WA, USA).

### 2.5. Wound Healing Assay

Wound healing assay was used for evaluate cell migration capacity. MCF-7 and MDA-MB-231 cells were seeded in six well plates (Nest Biotechnology, China) and incubated. At full confluence, the monolayer was scratched along a straight line using a sterile 10 μL pipette tip (*n* = 6). During the wound-healing assay, the photographed areas were pre-marked at the bottom of each well in 6-well plates, and images were consistently captured from the same fields at 0, 24, and 48 h. The wound area was photographed at 0, 24, and 48 h (ZOE, Tokyo, Japan). The distance between the wound edges was measured using ImageJ 1.54g software. Relative migration (%) was normalized to 0 h distance.

### 2.6. Evaluation of Apoptosis with Annexin-V/PI

Apoptosis was evaluated by using Annexin V-FITC apoptosis detection kit (eBio-science- Invitrogen, Thermo Fisher Scientific, Brunn am Gebirge, Austria). MCF-7 and MDA-MB-231 cells were seeded (4 × 10^5^ cells/well) into six well plates and incubated. After reaching ~60% confluence cells were treated with 400 μM carvacrol, and maintained for 48 h. To determine apoptosis, carvacrol-treated groups and non-treated control groups were washed twice with cold PBS (pH: 7.4). The supernatant was discarded, and 0.5 mL of PBS was added on the pellets. 100 µL of cell suspension was added to flow tubes for each sample. Annexin V-FITC and PI were added and incubated for 20 min in the dark. After that, 400 µL of Annexin buffer solution was added and read on the device (*n* = 3). Data were analyzed using the Kaluza analysis program using a flow cytometer (Beckman Coulter, S Kraemer Blvd, CA, USA). Data were analyzed using standard four-quadrant gating. Before the study, parameters were adjusted to create a template suitable for apoptosis.

### 2.7. Reactive Oxygen Species (ROS) Activity

The 2′-7′-Dichlorodihydrofluorescein diacetate assay (DCFH-DA) (Sigma Aldrich, Darmstadt, Germany), which directly measures the redox status of cells, was used to assess changes in intracellular reactive oxygen species (ROS) levels. When cells became at ~60% confluence, cells were treated with 400 μM carvacrol, and re-incubated. After 48 h, cells were washed with PBS and incubated with DCFH-DA (10 μM) for 30 min at 37 °C. 10 μL of DCFH-DA (Sigma Aldrich) was added to each tube. The cells were incubated in a water bath at 37 °C for 30 min. Samples were read using a Beckman Coulter Navios flow cytometer (Navios 3L10) (Beckman Coulter, USA) at 485 nM excitation and 535 nM emission wavelengths (*n* = 3). Data was analyzed with the Kaluza analysis program (2.1 version) and recorded as a histogram. The mean fluorescence intensity values of the cells were used in statistical analyses.

### 2.8. Analysis of CD44/CD133 (Cancer Stem Cell Marker) by Flow Cytometry

Forty-eight-hour carvacrol-treated and control cells were washed with PBS and centrifuged (Hettich ROTOFIX 32 A, Tuttlingen, Germany) at 1800 rpm for 5 min. The pellets were resuspended in 500 µL PBS, and 100 µL of each suspension was incubated with 5 µL PB-conjugated CD44 and 5 µL APC-conjugated CD133 antibodies (Immunotech SAS, Marseille, France) for 15 min in the dark. After staining, cells were washed with PBS and analyzed on a Beckman Coulter Navios flow cytometer (Navios 3L10) (Beckman Coulter, USA) (*n* = 2). Fluorescence signals were recorded and quantified using Kaluza software (2.1 version).

### 2.9. Quantitative Real Time Polymerase Chain Reaction (qPCR)

Cells were seeded in 6-well plates at a density of 4 × 10^5^ cells per well. After 24 h, cells were treated with the IC_50_ concentration of carvacrol, while untreated cells served as the control group. Following 48 h of incubation, total RNA was isolated using the EZ-10 RNA Miniprep Kit (Biobasic, Markham, ON, Canada) according to the manufacturer’s instructions. RNA concentration and purity were measured using a nanodrop spectrophotometer (Thermo Fisher Scientific, Waltham, MA, USA). Complementary DNA (cDNA) was synthesized from 600 ng of total RNA using the OneScript^®^ Plus cDNA Synthesis Kit (Applied Biological Materials, Richmond, BC, Canada).

Quantitative real-time PCR (qPCR) was performed using BlasTaq™ 2 × qPCR Mas-terMix (Applied Biological Materials, Richmond, Canada) on a qPCR (Bio-Rad, Hercules, CA, USA) system under the following conditions: initial denaturation at 95 °C for 3 min, followed by 45 cycles of 95 °C for 15 s, 60 °C for 15 s, and 72 °C for 15 s. Each reaction contained 2 µL cDNA, 0.5 µL of each primer (10 µM), and 10 µL of MasterMix in a total volume of 20 µL. Primer sequences are listed in Table 1. *GAPDH* was used as the reference gene, and relative expression levels were calculated using the 2^−ΔΔCt^ method. All reactions were performed in duplicate (*n* = 2).

### 2.10. Statistical Analysis

All statistical analyses were performed using GraphPad Prism 10.5.0. Data are presented as mean ± SD. Differences between groups were analyzed using Student’s *t*-test or one-way/two-way ANOVA followed by Tukey’s post hoc test, as appropriate. Fisher’s exact test was applied for categorical variables. A *p* < 0.05 was considered statistically significant.

## 3. Results

### 3.1. Differential Expression and Survival Analysis of Apoptosis and Resistance Related Genes in BC

**Figure 1:**
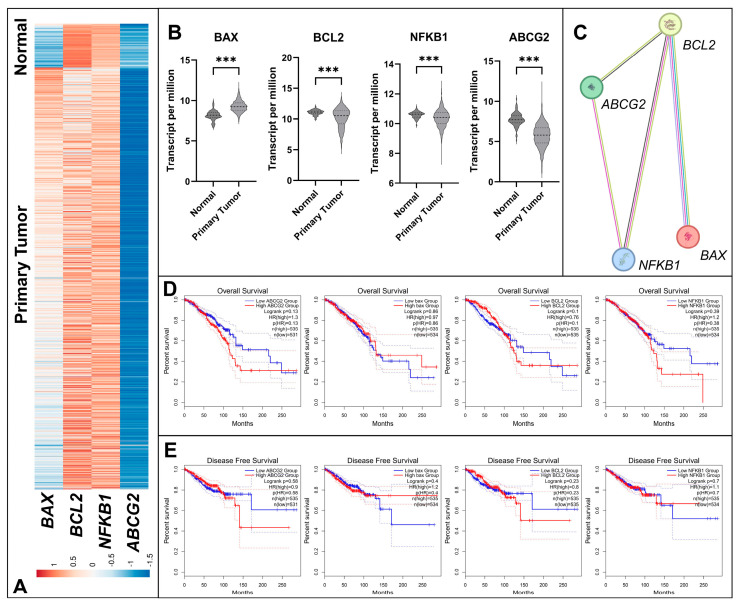
Integrated bioinformatics analysis of *BAX*, *BCL2*, *NF_K_B1*, and *ABCG2* genes in breast tumor tissue samples. (**A**) Heatmap of gene expression levels in normal breast tissues (*n* = 114) and primary breast tumor tissues (*n* = 1097) based on TCGA-BRCA data. (**B**) Violin plots showing the differential expression of the four genes between normal and tumor tissues, with statistical comparisons performed using the Mann–Whitney *U* test (*** *p* < 0.001). All four genes were upregulated in tumor tissues compared with normal tissues. (**C**) PPI network of *BAX*, *BCL2*, *NF_K_B1*, and *ABCG2* constructed using the STRING database (Nodes: 4; Edges: 4; Average node degree: 2; Average local clustering coefficient: 0.833; Expected edges: 1; PPI enrichment *p* = 0.018), indicating a significant functional interaction among these proteins. (**D**) Kaplan–Meier overall survival analysis of BC patients using median expression as cutoff: *ABCG2* (*p* = 0.13), *BAX* (*p* = 0.86), *BCL2* (*p* = 0.10), *NF_K_B1* (*p* = 0.13). (**E**) Kaplan–Meier disease-free survival analysis: *ABCG2* (*p* = 0.58), *BAX* (*p* = 0.40), *BCL2* (*p* = 0.23), *NF_K_B1* (*p* = 0.70). TCGA-BRCA data were utilized for survival analyses, and correlations were evaluated using GEPIA2.

*BAX*, *BCL2*, *NF_K_B1* and *ABCG2* genes expression were compared by using breast tumor tissues on TCGA-BRCA (Figure 1). When the 1097 tumor samples were compared with 114 normal tissue samples (Figure 1A), it was shown that *BAX* gene had enhanced expression profile while *BCL2* had lower median (Figure 1B) in tumor tissues. Also, *NF_K_B1* and *ABCG2* genes show decreased pattern in BC patients when compared with normal tissue samples (Figure 1B).

Using the STRING database, a stronger relationship was observed in the potential PPI analysis between the *BAX*, *BCL2*, *NF_K_B1*, and *ABCG2*, indicating a significant functional interaction among these proteins (*p* = 0.018) (Figure 1).

Survival outcomes of BC patients were further assessed by stratifying patients into low and high expression groups based on median gene expression levels. Kaplan–Meier overall survival (OS) analysis revealed no significant associations for *ABCG2* (*p* = 0.13), *BAX* (*p* = 0.86), *BCL2* (*p* = 0.10), or *NF_K_B1* (*p* = 0.13) (Figure 1D). Similarly, disease-free survival (DFS) analysis showed no statistically significant differences for *ABCG2* (*p* = 0.58), *BAX* (*p* = 0.40), *BCL2* (*p* = 0.23), or *NF_K_B1* (*p* = 0.70) (Figure 1E). These results indicate that, although these genes exhibit differential expressions between normal and tumor tissues, their expression alone does not appear to serve as an independent prognostic factor in BC.

### 3.2. Carvacrol Suppresses Proliferation and Migration in Both HR+ and TNBC Cells

**Figure 2:**
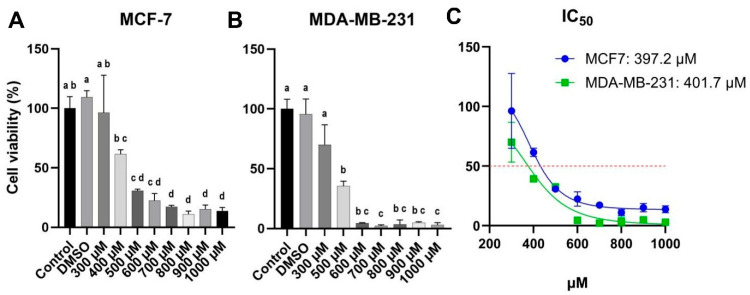
Effects of carvacrol on the viability of MCF-7 and MDA-MB-231 BC cells. (**A**) MCF-7 cells viability after 48 h of carvacrol treatment. (**B**) MDA-MB-231 cells viability after 48 h of carvacrol treatment. (**C**) IC_50_ values were determined as 397.2 µM for MCF-7 and 401.7 µM for MDA-MB-231 cells. Based on the IC_50_ values, BC cells were treated with 400 µM carvacrol to further assess its effect on cell viability. The DMSO group was considered as vehicle control. Different letters indicate statistically significant differences in panel (**A**,**B**) (Post Hoc Tukey test).

BC cells were treated with varying concentrations of carvacrol (300–1000 µM) for 48 h (Figure 2). In both cell lines, carvacrol treatment at approximately 400 µM led to nearly a 50% reduction in cell viability, yielding comparable IC_50_ values of around 400 µM (397.2 µM for MCF-7 and 401.7 µM for MDA-MB-231) (Figure 2). Cells in the DMSO group were treated with the same concentration of DMSO (1.5:1000) as in the highest carvacrol treatment group (1000 µM carvacrol group). No statistical difference was observed between the control and DMSO groups; thus, the DMSO group was excluded from further analyses.

**Figure 3:**
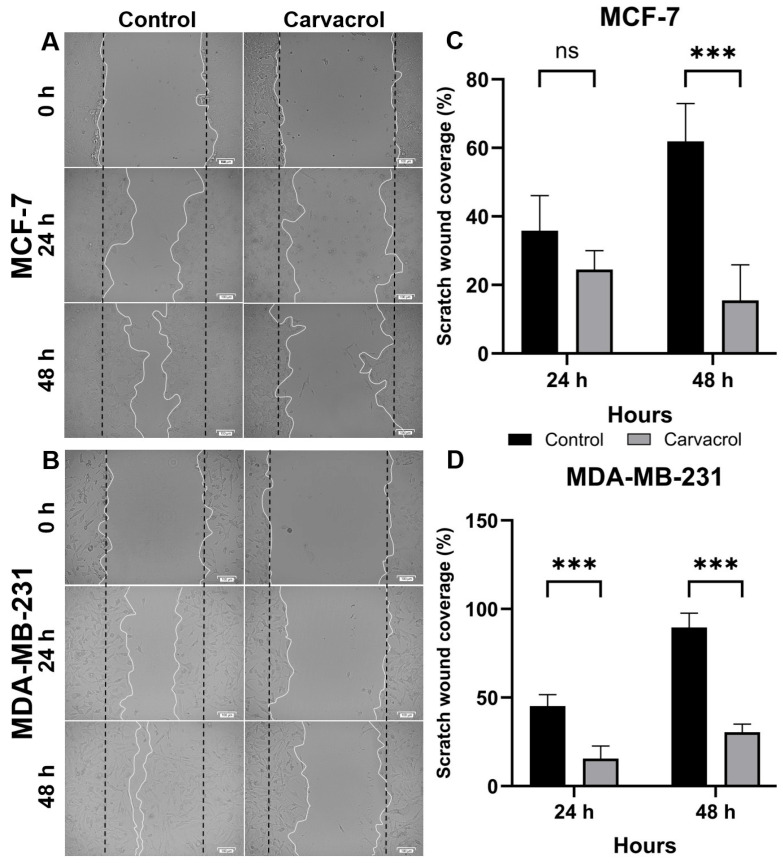
Anti-migratory effect of carvacrol on MCF-7 and MDA-MB-231 cells. Wound healing assays were performed by creating scratches at 0 h, followed by treatment with 400 µM carvacrol. Representative images of wound closure at 0 h, 24 h, and 48 h are shown for MCF-7 (**A**) and MDA-MB-231 (**B**) cells. Quantification of wound closure rates was performed at 24 h and 48 h using the calibration bars (calibration bar = 100 µm) in the images, with the scratch distance at 0 h considered as 100% (*t*-test: *** *p* < 0.001; ns: not significant). Quantification of scratch wound closure (%) is shown for MCF-7 (**C**) and MDA-MB-231 (**D**) cells.

The anti-migratory effect of carvacrol on MCF-7 and MDA-MB-231 BC cells were also evaluated. The distances between the scratched lines after 24 h and 48 h of carvacrol treatment are presented in Figure 3A,B. At 24 h, carvacrol did not significantly inhibit the wound healing ability of MCF-7 cell line compared with the control group, although it reduced wound closure by more than two-fold in MDA-MB-231 cells (Figure 3C,D). At 48 h, however, carvacrol significantly suppressed the wound healing ability of both cell lines relative to the control group. Therefore, further investigations were conducted at 48 h of carvacrol treatment.

### 3.3. Carvacrol Induces Apoptosis via Modulation of BAX/BCL2 Ratio

**Figure 4:**
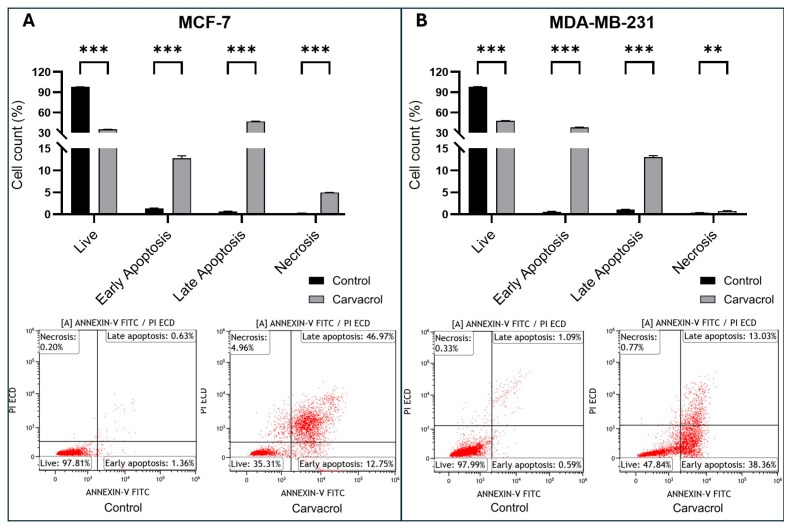
Induction of apoptosis by carvacrol in (**A**) MCF-7 and (**B**) MDA-MB-231 cells. Cells were treated with 400 µM carvacrol for 48 h, and apoptosis was evaluated by Annexin V/PI staining using flow cytometry. The proportions of live, early apoptotic, late apoptotic, and necrotic cells were determined. Panel (**A**) shows representative results for MCF-7 cells, while Panel (**B**) shows those for MDA-MB-231 cells. Carvacrol treatment effectively induced apoptosis in both BC cell lines (*t*-test: ** *p* < 0.01, *** *p* < 0.001).

The effect of carvacrol on apoptosis in MDA-MB-231 and MCF-7 cells was examined. Cells were treated with carvacrol for 48 h. The results showed that carvacrol induced cell apoptosis (Figure 4). The viability rate of MCF-7 control cells was 97.81%. The survival rate of MCF-7 cells treated with carvacrol for 48 h was observed as 35.31%. The rates of early apoptosis were 1.36% in MCF-7 control cells. As a result of treatment with carvacrol, this rate showed a high expression of 12.75%. The rates of late apoptosis were 0.63% in the MCF-7 control. In MCF-7 treated with carvacrol, the rate increased to 46.97% (Figure 4A).

Likewise, the live rate of MDA-MB-231 control cells was 97.99% (Figure 4B). The survival rate of MDA-MB-231 cells treated with carvacrol for 48 h dropped to 47.84%. Early apoptosis rates were 0.59% in control group of MDA-MB-231 cells. As a result of treatment with carvacrol, this rate increased to 38.36%. Late apoptosis rates were 1.09% in the MDA-MB-231 control group. The rate increased to 13.03% in MDA-MB-231 treated with carvacrol.

### 3.4. Carvacrol Reduces Intracellular ROS Levels in a Subtype-Dependent Manner

**Figure 5:**
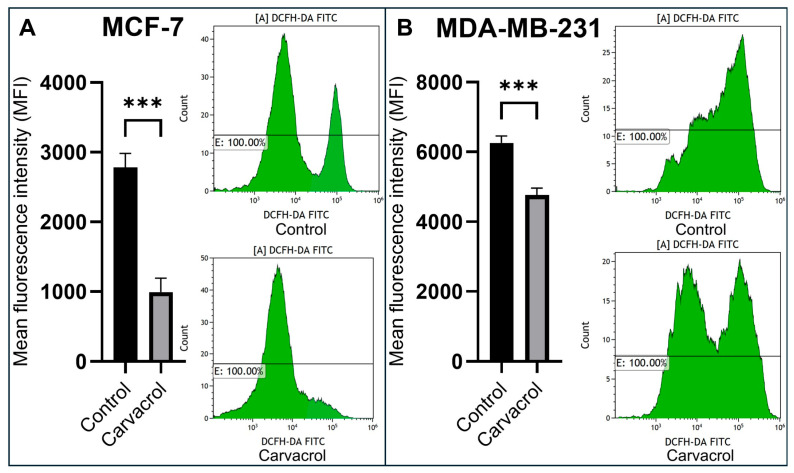
Intracellular ROS production in MCF-7 and MDA-MB-231 cells treated with 400 µM carvacrol. Flow cytometry analysis was performed after 48 h. Carvacrol caused a marked decrease in ROS production in MCF-7 cells (**A**), whereas a more moderate reduction was observed in MDA-MB-231 cells (**B**) (*t*-test: *** *p* < 0.001).

Intracellular ROS levels in carvacrol-treated MCF-7 and MDA-MB-231 BC cells were also evaluated by flow cytometry using by DCFH-DA probe. Compared with the control group, carvacrol treatment for 48 h led to a pronounced decrease in ROS levels in MCF-7 cells (~2.8-fold) (Figure 5A), whereas the reduction in MDA-MB-231 cells was more moderate (~1.3-fold) (Figure 5B), with approximately a 20% decrease observed. This differential response may reflect the distinct redox regulation and metabolic profiles of the two cell lines. The overall decline in intracellular ROS levels indicates that carvacrol may modulate oxidative stress as part of its anticancer activity, consistent with its previously reported antioxidant effects.

### 3.5. Carvacrol Downregulates CD44^+^ Stemness Marker in Both Subtypes

**Figure 6:**
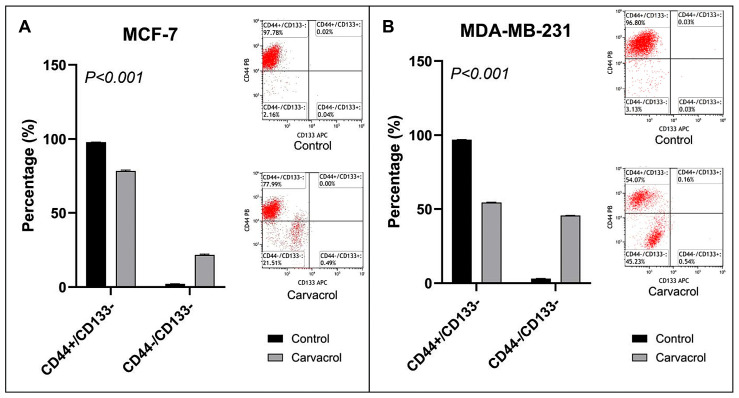
Effect of carvacrol on CD44 and CD133 expression in MCF-7 and MDA-MB-231 cells. Flow cytometry analysis was performed to evaluate changes in cancer stem cell markers after 48 h of treatment with 400 µM carvacrol. In control MCF-7 cells, CD44^+^/CD133^−^ expression was 97.78%, which decreased to 77.99% following carvacrol treatment, while no change was observed in CD133 expression (**A**). Similarly, control group of MDA-MB-231 cells exhibited 96.8% CD44^+^/CD133^−^ expression, which was reduced by 54.07% after carvacrol treatment, whereas CD133 expression remained unchanged (**B**). To compare CD44 and CD133 expressions, Fisher’s Exact Test was performed.

An investigation was conducted to observe the change in cancer stem cell markers in the MCF-7 cancer cell line (Figure 6A). CD44^+^/CD133^−^ expression was observed as 97.78% in control MCF-7 cells. In MCF-7 cells treated with carvacrol for 48 h, this expression was found to be 77.99%. While there was a decrease in the CD44^+^ cancer stem cell marker, no change was detected in the other marker, CD133 expression.

MDA-MB-231 cells were also expressed at a significant level of CD44 and CD133 proteins (Figure 6B). CD44^+^/CD133^−^ expression was observed as 96.8% in control MDA-MB2-31 cells. It was noteworthy that the expression was found to be 54.07% lower in MDA-MB-231 cells treated with carvacrol for 48 h. While there was a decrease in the CD44^+^ cancer stem cell marker, there was no change in CD133 expression like the MCF-7 cell line.

### 3.6. Subtype-Specific Modulation of BAX, BCL2, NF_K_B1, and ABCG2 Expression

**Figure 7:**
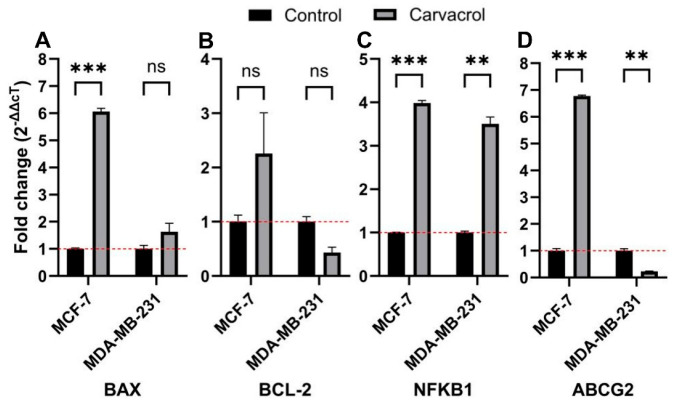
Relative gene expression of *BAX* (**A**), *BCL2* (**B**), *NF_K_B1* (**C**) and *ABCG2* (**D**) genes in MCF-7 and MDA-MB-231 BC cells treated with 400 µM carvacrol for 48 h. Fold change of gene expressions were calculated with 2^−ΔΔCt^ method (*t*-test: ** *p* < 0.01, *** *p* < 0.001; ns: not significant). A dotted line was added at y = 1 to indicate the normalized mean expression level of the control group.

Expressions of *BAX*, *BCL2*, *NF_K_B1* and *ABCG2* genes after treatment of 400 µM carvacrol were also investigated in MCF-7 and MDA-MB-231 BC cell lines (Figure 7). *BAX*, a pro-apoptotic gene, and *BCL2*, an anti-apoptotic gene, are considered key markers of apoptotic stimuli. The expression ratio (BAX/BCL2) reflects the apoptotic tendency of cells. In our results, treatment with 400 µM carvacrol increased the *BAX*/*BCL2* ratio to 2.7 in MCF-7 and 4.0 in MDA-MB-231 cells. These findings indicate that carvacrol induces mitochondria-related apoptosis. On the other hand, carvacrol treatment increased *NF_K_B1* gene, a pleiotropic transcription factor, expression in both cell lines. The *ABCG2* gene, also known as the BC resistance protein, encodes a drug efflux pump and is a key member of the ABC transporter superfamily. Following carvacrol treatment, we observed a marked increase in *ABCG2* expression in MCF-7 cells, whereas MDA-MB-231 cells showed a decreasing trend.

## 4. Discussion

This study aimed to evaluate the anticancer potential of carvacrol in two BC cell lines representing distinct phenotypes: MCF-7 and MDA-MB-231. While MCF-7 cells display a HR^+^ BC phenotype, MDA-MB-231 cells represent a TNBC phenotype [25]. These BC phenotypes differ in prognosis and require distinct therapeutic strategies in conventional treatment regimens [26]. Nevertheless, the search for anticancer agents effective across different cancer subtypes remains a critical field of investigation. Plants, with their diverse natural compounds, have historically contributed significantly to the development of pioneering drug candidates [27,28,29]. Carvacrol is one such phytochemical, reported to possess various anticancer properties [30,31]. Previous studies demonstrated that carvacrol disrupts mitochondrial membrane potential [32], and promotes cytochrome-c release, thereby triggering the activation of pro-apoptotic caspase-3 and caspase-9, as well as PARP cleavage, another hallmark of apoptosis [33]. Thus, carvacrol has attracted considerable attention as a mitochondria-targeting agent capable of inducing programmed cell death.

Our cell-viability results revealed that carvacrol exhibited comparable IC_50_ values in MCF-7 and MDA-MB-231 cells (Figure 2C), with IC_50_ values of 397.2 µM and 401.7 µM, respectively. These closely aligned IC_50_ values suggest that carvacrol may induce apoptosis through a common pathway in both HR^+^ BC and TNBC phenotypes. The relatively high IC_50_ values (~400 µM) observed in this study may be attributed to differences in experimental setup and compound handling reported across previous studies. Moreover, carvacrol’s volatility and low solubility could limit its effective cellular uptake, resulting in higher apparent IC_50_ values despite consistent biological activity. Apoptosis is a key mechanism by which cancer therapies exert cytotoxicity, and nearly all conventional anticancer drugs are known to induce programmed cell death [34]. As the central hub of energy production in eukaryotic cells, mitochondria play a pivotal role in apoptosis through their regulation of pro-apoptotic and anti-apoptotic proteins [35]. Like many apoptotic agents, carvacrol has been shown to modulate mitochondrial proteins associated with apoptosis [32]. The balance between the pro-apoptotic *BAX* and the anti-apoptotic *BCL2* genes is a key determinant of mitochondrial integrity, influencing the zymogen caspase cascade and subsequent protease/endonuclease-driven DNA fragmentation [36,37]. Clinically, the *BAX*/*BCL2* ratio is considered an indicator of apoptotic susceptibility, with an increased ratio reflecting greater sensitivity to apoptosis [38,39].

In our experiments, treatment with 400 µM carvacrol for 48 h significantly increased the *BAX*/*BCL2* expression ratio compared to controls in both cell lines (Figure 7). Notably, this increase was ~2.7-fold in MCF-7 cells and ~4.0-fold in MDA-MB-231 cells. This difference may reflect intrinsic variations in the phenotypes of the two BC lines. Interestingly, carvacrol induced a greater increase in the *BAX*/*BCL2* ratio in the TNBC line, which is associated with a poorer prognosis, highlighting its potential anticancer relevance. Flow cytometry further revealed that, following carvacrol treatment, 12.75% of MCF-7 cells were in early apoptosis and 46.97% in late apoptosis (Figure 4A). By contrast, 38.36% of MDA-MB-231 cells were in early apoptosis and only 13.03% in late apoptosis (Figure 4B). This trend appears consistent with the observed differences in *BAX*/*BCL2* ratios, as MCF-7 cells showed a ~4.5-fold higher proportion of late-apoptotic cells compared to MDA-MB-231 under identical conditions. Collectively, these data suggest that carvacrol triggers apoptosis more rapidly in MCF-7 cells, while inducing a delayed apoptotic response in MDA-MB-231 cells. Given that the *BAX*/*BCL2* ratio is linked to the early stages of apoptosis and PI incorporation requires disruption of membrane integrity [40], these findings support the notion that apoptotic induction by carvacrol in TNBC cells occurs at later stages compared to HR^+^ BC cells. Although qPCR results demonstrated transcriptional modulation of BAX and BCL2, their protein-level regulation has already been widely validated in the literature, supporting their established roles in apoptosis induction.

While many plant-derived agents exhibit strong antioxidant activity, this feature can sometimes hinder their suitability as anticancer agents due to potential interference with conventional therapies [11,12]. Apoptosis may be initiated either through death receptor signaling or via mitochondrial stress [41]. In the mitochondrial stress pathway, intracellular stressors such as ROS and Ca++ promote the release of cytochrome-b and AIF from mitochondria, triggering caspase-mediated apoptotic cascades [42]. Bax, a pro-apoptotic protein encoded by the BAX gene, can interact with plant-derived polyphenolic compounds, and these interactions may alter its conformation, thereby modulating apoptosis [43]. Mari A. et al. reported that carvacrol treatment of MCF-7 cells significantly inhibited PI3K/p-AKT protein expression, which in turn led to the induction of apoptosis by decreasing Bcl2 and increasing Bax protein expression. Furthermore, in this study, Annexin V/PI staining demonstrated that carvacrol induced apoptosis in MCF-7 cells via the PI3K/Akt signaling pathway [19]. Another study reported that combining carvacrol with 5-FU significantly increased apoptosis in MCF-7 cells [44]. Similarly, Moradipour et al. showed that carvacrol inhibited MCF-7 cell growth and induced p53-dependent apoptosis via the Bax/Bcl-2 pathway [31]. Additional evidence in MDA-MB-231 cells further confirmed the antitumor activity of carvacrol, underscoring its therapeutic potential in BC [31]. A 2025 study by Dariushnejad et al. demonstrated that carvacrol exerts anticancer effects and shows strong synergy with paclitaxel, markedly reducing paclitaxel dosage requirements. Combination treatment induced high cell death rates and regulated the apoptotic axis (Bax/Bcl-2/p53) [45]. In 2021, Li et al. reported that carvacrol dose-dependently inhibited viability in multiple BC cell lines (BT-483, BT-474, MCF-7, MDA-MB-231, MDA-MB-453) and suppressed TRPM7 channel activity (IC_50_ ≈ 83.8 μM), with marked inhibition at 200 μM. At this concentration, carvacrol induced G_1_/G_0_ arrest and cyclin/CDK regulation, while TRPM7 knockdown abrogated its effects in MDA-MB-231 cells, indicating a partially TRPM7-mediated mechanism with enhanced apoptosis at ≥300 μM [18]. Hu-hu Chen and Siat Yee Fong demonstrated that carvacrol inhibited proliferation of HCC1937 BC cells (IC_50_ = 320 µM, 24 h) by inducing G0/G1 arrest and Annexin V-FITC/PI flow cytometry revealed a marked, dose-dependent elevation in apoptotic cells. Western blot analyses further showed decreased Bcl-2 with increased Bax, cytochrome c, and caspase-3, indicating activation of the mitochondrial apoptosis pathway [46].

Carvacrol, a phenolic compound, has been reported to possess both anticancer and antioxidant properties [47,48]. In line with this, we found that 400 µM carvacrol treatment for 48 h reduced total ROS levels by ~2.8-fold in MCF-7 cells and ~1.3-fold in MDA-MB-231 cells, indicating phenotype-specific antioxidant effects. At the same time, carvacrol increased *BAX* expression by ~6.0-fold in MCF-7 and ~1.5-fold in MDA-MB-231, while increasing *BCL2* expression by ~2.3-fold in MCF-7 but reducing it by ~0.4-fold in MDA-MB-231 (Figure 5). These findings suggest that, despite its antioxidant effects, carvacrol modulates the *BAX*/*BCL2* ratio in a manner that contributes to apoptotic induction in BC cells. Nevertheless, given its dual pro-apoptotic and strong antioxidant activities, the in vivo anticancer efficacy of carvacrol warrants further investigation. In vitro study by Khan et al. have shown that carvacrol exerts dose- and time-dependent cytotoxicity, apoptosis induction, and cell-cycle arrest in various carcinoma cell lines, including BC. These effects involve modulation of apoptotic genes (downregulation of *BCL2*, upregulation of *BAX*, and *caspase-3/6/9*), regulation of pathways such as NF-κB, and modulation of ROS production [17]. Imran et al. showed that carvacrol treatment significantly reduced ROS levels, particularly in MDA-MB-231 cells. This suggests that carvacrol’s antioxidant activity plays a critical role in its cytotoxic effects on BC cells [49].

Beyond apoptosis and ROS activity, the effectiveness of anticancer agents also depends on their ability to target cancer stem cell marker levels. If cancer stem-like cells are not adequately suppressed, tumors may relapse aggressively. CD44 and CD133 are widely recognized markers for identifying cancer stem cells [50,51]. Our results demonstrated that carvacrol reduced the CD44^+^ levels by ~22% in MCF-7 cells and nearly by half in MDA-MB-231 cells (Figure 6). Since CD44 is a membrane adhesion molecule [52], its suppression suggests that carvacrol may impair adhesion, migration, invasion, and metastasis of BC cells. Supporting this, wound-healing assays revealed a marked reduction in migration in both cell lines following treatment (Figure 3). However, double-positive (CD44^+^/CD133^+^) levels were absent in both lines (Figure 6), leaving the impact of carvacrol on CD133^+^ cells inconclusive. According to the study by Mao et al., the MCF-7 cell line had low CD133 expression and cancer stem cell characteristics. In our study, we did not observe CD133 expression in MCF-7 and MDA-MB-231 cell lines [53]. This lack of detectable CD133 signal may be related to epitope masking or glycosylation-dependent variation in the AC133 epitope, as previously reported [54,55]. Such post-translational and methodological factors can lead to underestimation of CD133^+^ populations in flowcytometric analyses, particularly under adherent culture conditions.

*NF_K_B1* encodes a subunit of the *NF-_K_B* transcription factor complex, a master regulator of inflammation, immunity, and cell proliferation. Aberrant *NF-_K_B* signaling is central to BC progression, therapeutic resistance, and metastasis [56]. Carvacrol has been suggested to exert anticancer activity, at least in part, via inhibition of *NF-_K_B* signaling [57,58]. However, our results demonstrated increased *NF_K_B1* expression in both cell lines after carvacrol treatment (Figure 7). This discrepancy may stem from differences between transcriptional and translational regulation. While prior studies reported inhibition of *NF-_K_B* protein expression by carvacrol [57,59], our findings raise the possibility that carvacrol may induce *NF_K_B1* mRNA expression. The observed elevation in *NF_K_B1* expression may represent a compensatory transcriptional response to oxidative or apoptotic stress rather than functional activation of the *NF-_K_B* pathway

Another critical mechanism of therapy resistance involves efflux of therapeutic agents by membrane transporters. *ABCG2*, a member of the ABC superfamily, is a major contributor to drug resistance in cancer [60,61]. Together with transporters such as *ABCA1* and *ABCB1*, *ABCG2* plays a key role in BC chemoresistance. Overexpression of *ABCG2* is linked to poor chemotherapy response and adverse prognosis [62]. For instance, Zhang et al. reported higher *ABCG2* expression in ER^+^ BC patients than in TNBC or HER2^+^ subtypes, with elevated *ABCG2* correlating with larger tumor size, lymphatic spread, and poor pathological response [63]. Conversely, Nedeljković et al. observed that TNBC tumors often display higher *ABCG2* expression, and in some cases, elevated levels were associated with less aggressive behavior and improved survival, suggesting a complex and context-dependent role of *ABCG2* [64]. In our study, carvacrol upregulated *ABCG2* expression by ~6.7-fold in MCF-7 (HR^+^ BC) cells, whereas it downregulated *ABCG2* expression by ~0.2-fold in MDA-MB-231 (TNBC) cells (Figure 7). The differential regulation of *ABCG2* between MCF-7 and MDA-MB-231 cells suggests subtype-specific effects of carvacrol on multidrug resistance mechanisms. The observed downregulation in TNBC cells may reflect a potential sensitizing effect, whereas the mild increase in HR^+^ cells could indicate a compensatory metabolic adjustment. Functional assays assessing drug-efflux capacity or combination therapy outcomes are required to clarify whether carvacrol can effectively modulate resistance pathways.

Recent preclinical studies have evaluated carvacrol in combination with chemo-therapeutic agents, showing synergistic cytotoxicity and apoptosis induction in animal tumor models [65,66,67]. However, no clinical trials are currently available, highlighting the need for further in vivo validation. Given these promising yet preliminary outcomes, recent research has also focused on developing novel formulation strategies to improve carvacrol’s pharmacological performance by enhancing its solubility, stability, and targeted delivery efficiency. Formulation strategies such as nanoparticle encapsulation and quantum dot-based delivery have been shown to improve carvacrol’s physicochemical stability and targeted bioavailability, representing promising approaches for its future therapeutic use. Akhlaq et al. developed carvacrol-loaded chitosan nanoparticles (~80 nm, +24.7 mV) with high entrapment efficiency and pH-responsive release, which were non-toxic to Vero cells but strongly inhibited MCF-7 and HeLa growth, especially in combination with doxorubicin or ciprofloxacin. Synergistic effects (CI: 0.31–0.34, high dose-reduction indices) indicated enhanced apoptosis and cell-cycle arrest, highlighting nanoparticles as a strategy to boost carvacrol’s bioavailability and therapeutic potential [68]. Srinivasan et al. synthesized pH-sensitive carvacrol-loaded ZnO quantum dots (CVC-ZnO QDs), which showed pH-dependent release and strong cytotoxicity against MDA-MB-231 cells (IC_50_: 20.47 µg/mL), inducing ROS generation, mitochondrial dysfunction, lipid peroxidation, and apoptosis (~49–91%). Molecular analyses revealed decreased *BCL-2* with increased *BAX*, caspase-9, and caspase-3, indicating mitochondria-mediated apoptosis, though the absence of in vivo data remains a limitation [69].

In summary, this preliminary study provides novel insights into the molecular and cellular mechanisms by which carvacrol may exert its anticancer effects in BC. By demonstrating its ability to modulate key apoptotic regulators, reduce oxidative stress, and suppress cancer stem cell markers levels, our findings contribute to the growing body of evidence supporting phytochemicals as potential therapeutic agents in oncology, especially in BC. Importantly, these results underscore the potential of carvacrol to act not only as a direct cytotoxic agent but also as a modulator of pathways associated with drug resistance and tumor progression. Ongoing investigations into carvacrol’s role in BC are therefore vital for enhancing therapeutic efficacy, developing novel combinational strategies with conventional chemotherapeutics, and ultimately improving patients’ quality of life. Collectively, these findings highlight carvacrol’s therapeutic promise; however, validation with in vivo models and clinical cohorts remains crucial to confirm the translational relevance of our observations and to establish its safety and efficacy profile in the clinical setting.

## 5. Conclusions

This study demonstrates that carvacrol exerts multi-targeted anticancer effects in both HR^+^ (MCF-7) and TNBC (MDA-MB-231) BC cells by enhancing the *BAX*/*BCL2* ratio, inducing apoptosis, reducing oxidative stress, and downregulating CD44^+^ cancer stem cell marker levels. Importantly, we reveal for the first time the sub-type-specific modulation of the *ABCG2* drug resistance gene, suggesting that carvacrol may sensitize TNBC cells to therapy while exerting protective effects in HR^+^ cells. These results highlight carvacrol as a promising phytochemical candidate for the development of subtype-tailored BC therapeutics. Further in vivo and clinical studies are warranted to validate these findings and establish its safety and translational efficacy. Beyond the pathways discussed here, the interaction between ROS modulation and stemness regulation represents a promising but underexplored mechanism that may contribute to carvacrol’s subtype specific effects. Furthermore, comprehensive in vivo pharmacokinetic and toxicity evaluations are warranted to assess its safety, metabolic stability, and therapeutic potential under physiological conditions.

## Figures and Tables

**Table 1:** Forward and reverse primer sequences of the studied genes.

Gene	Forward Primer	Reverse Primer
*BAX1*	5′-CTACAGGGTTTCATCCAG-3′	5′-CCAGGAGAAATCAAACAGAG-3′
*BCL2*	5′-GTGGATGACTGAGTACCT-3′	5′-CCAGGAGAAATCAAACAGAG-3′
*NF_K_B1*	5′-TACGATGGAACCACACCCCTG-3′	5′-TCTGCTCCTGCTGCTTTGAGA-3′
*ABCG2*	5′-AGCAGCAGGTCAGAGTGTGG-3′	5′-GATCGATGCCCTGCTTTACC-3′
*GAPDH*	5′-CCACCCATGGCAAATTCC-3′	5′-TGGGATTTCCATTGATGACAAG-3′

## Data Availability

The original contributions presented in this study are included in the article. Further inquiries can be directed to the corresponding author.

## Abbreviations

The following abbreviations are used in this manuscript:

BC	Breast cancer
TNBC	Triple-Negative Breast Cancer
BAX	Bcl-2 Associated X-protein
BCL2	B-Cell Lymphoma 2
NFKB	Nuclear Factor Kappa B Subunit 1
ABCG	ATP-Binding Cassette Sub-Family G Member 2
ABC	ATP-Binding Cassette
TCGA	The Cancer Genome Atlas
DMSO	Dimethyl Sulfoxide
CVDK8	Cell Viability Detection Kit 8
PI	Propidium Iodide
DCFH-DA	The 2′-7′-Dichlorodihydrofluorescein Diacetate Assay
ROS	Reactive Oxygen Species
qPCR	Quantitative Real Time Polymerase Chain Reaction
SD	Standard Deviation
OS	Overall Survival
DFS	Disease-Free Survival
